# The effect of Henna (*Lawsonia inermis*) vaginal suppository combined with antibiotic therapy in the treatment of cervicitis: An RCT

**DOI:** 10.18502/ijrm.v22i4.16388

**Published:** 2024-06-12

**Authors:** Naeemeh Nabimeybodi, Fahimeh Nokhostin, Rahele Zareshahi, Mohammad Kamalinejad, Hedayat Akhundimeybodi, Farzan Madadizadeh, Mohsen Nabi Meybodi, Narges Seifi Mazraeno, Razieh Nabimeybodi

**Affiliations:** ^1^Department of Persian Medicine, School of Persian Medicine, Shahid Sadoughi University of Medical Sciences, Ardakan, Yazd, Iran.; ^2^Department of Obstetrics and Gynecology, Faculty of Medicine, Shahid Sadoughi University of Medical Sciences, Yazd, Iran.; ^3^Department of Pharmacognosy, School of Pharmacy, Shahid Sadoughi University of Medical Sciences, Yazd, Iran.; ^4^Traditional Pharmacy and Pharmaceutical Sciences Research Center, Faculty of Pharmacy, Shahid Sadoughi University of Medical Sciences, Yazd, Iran.; ^5^School of Pharmacy, Shahid Beheshti University of Medical Sciences, Tehran, Iran.; ^6^Department of Urology, School of Medicine, Shahid Bahonar Hospital, Kerman University of Medical Sciences, Kerman, Iran.; ^7^Center for Healthcare Data Modeling, Departments of Biostatistics and Epidemiology, School of Public Health, Shahid Sadoughi University of Medical Sciences, Yazd, Iran.; ^8^Department of Pharmaceutics, Faculty of Pharmacy, Shahid Sadoughi University of Medical Sciences, Yazd, Iran.; ^9^Department of Midwifery, Islamic Azad University, Meybod Branch, Yazd, Iran.

**Keywords:** Lawsonia inermis, Persian+traditional medicine, Uterine cervicitis, Infertility, Vaginal suppository, Clinical trial.

## Abstract

**Background:**

Cervicitis is a prevalent gynecologic disease, which does not usually respond to conventional treatments. Long-term cervicitis can cause serious health problems such as inflammation, infertility, and cancer. Henna oil, an herbal product in Persian medicine, is recommended for uterine diseases like cervicitis.

**Objective:**

This study aims to evaluate the efficacy of Henna oil as a vaginal suppository in combination with an antibiotic regimen in the treatment of cervicitis.

**Materials and Methods:**

This randomized placebo-controlled trial, included 92 non-menopausal women with cervicitis at the Baqaipur Clinic of Shahid Sadoughi hospital in Yazd and the Persian Medicine Health Center in Ardakan, Yazd, Iran. Participants were further divided into either the Henna oil vaginal suppository group or the placebo group (n = 46/each group). During the study, the antibiotic treatment was administered to both groups. Cervicitis symptoms were compared between the groups and within each group.

**Results:**

Of 92 included individuals, 41 in each group completed the study. Results revealed that significant differences were observed in some outcomes, including vaginal discharge (p < 0.001), cervical ulcer size (p < 0.001), dyspareunia (p = 0.046), and postcoital bleeding (p < 0.001), indicating that the treatment was more effective in the henna group compared to the placebo group.

**Conclusion:**

Findings supported that the vaginal suppository of Henna oil in combination with antibiotic therapy could be effective in the improvement of clinical symptoms of cervicitis regardless of its pathology.

## 1. Introduction 

Cervicitis is one of the prevalent female diseases, which is identified as inflammation of the cervix (1, 2). It can cause many complications like endometritis, pelvic inflammatory disease, chronic pelvic pain, damage and adhesion of tubal mucosa, ectopic pregnancy, infertility, and the progression of cervical cancer (2, 3). The prevalence of cervicitis among women referring to sexually transmitted diseases counseling clinics has been about 30–45%. The inflammation and infection of the cervix can be a risk factor for infertility. The women involved in cervicitis may not reveal any symptoms, but potential symptoms like purulent vaginal discharge and bleeding after intercourse or between menstrual periods could appear. In addition, some women may experience dysuria, vulvovaginal irritation, and dyspareunia. Typically, a purulent/mucopurulent endocervical discharge and/or cervical friability by light touching with a cotton swab is found in speculum examination (2).

Cervicitis may be caused by infectious or non-infectious agents. *Chlamydia trachomatis* and *Neisseria gonorrhoeae *arethe most common types that cause acute cervicitis (2, 4). Non-infectious agents that induce chronic cervicitis include mechanical stimulation or trauma caused by surgical instruments or devices such as diaphragm, condom, etc. (2). Of course, in about half of the cervicitis cases, the exact etiology is still unknown (5, 6).

Different therapeutic methods, including antibiotic therapy, surgery, cryotherapy, and cauterization, are applied for the improvement of cervicitis. However, there is no decisive efficacy despite the high costs and possible side effects such as cervical stenosis and infertility (7). Therefore, it is recommended that a novel remedy and treatment approach for cervicitis be found. Due to less invasion more economical, better effects, and fewer side effects, it is widely accepted and is progressing worldwide to employ complementary and alternative medicine modalities (8, 9). Iranian traditional medicine or Persian medicine (PM) is one of the complementary and alternative medicine methods dating back thousands of years (10). The symptoms of cervicitis can almost be matched with “*Qorhah-e-Rahem*” in PM (4). *Lawsonia inermis* Linn (Family: Lythraceae), commonly known as Henna, is an important native medicinal plant in PM (9).

In PM resources like the Canon of Medicineof Avicenna recommend the use of Henna oil as a vaginal form for the treatment of various uterine diseases, including *Qoruhe-e-Rahem *(9, 11)*. *Henna possesses some pharmacological properties, including wound healing, antifungal, antioxidant, antibacterial, anti-ulcer, anti-inflammatory, and anti-cancer effects (12), which can be beneficial in cervicitis. The preparation of the Henna oil vaginal suppository method was previously standardized (9).

This randomized study was conducted to evaluate the efficacy of the Henna oil suppository as an herbal product to treat cervicitis among women compared to a placebo, while all participants received antibiotic therapy.

## 2. Materials and Methods

### Study design

This study was designed as a randomized clinical trial to investigate the efficacy of the Henna oil vaginal suppository on women with confirmed cervicitis in comparison with a placebo. It was performed at the Baqaipur Clinic of Shahid Sadoughi hospital in Yazd and the Persian Medicine Health Center in Ardakan, Yazd, Iran, from August 2021 to October 2021.

### Study participants

The study's inclusion criteria encompassed married non-menopausal women aged 21–50 yr with established cervicitis diagnosed via colposcopy and biopsy, displaying symptoms such as refractory vaginal discharge, postcoital bleeding (PCB), abnormal uterine bleeding, abnormal pap smear results, or abnormal cervix appearance during speculum examination. Exclusion criteria involved pregnant or lactating individuals, those with any addiction, presence of herpes sores or genital warts upon examination and/or positive human papillomavirus test, cervicitis dysplasia or cancer, recent use of medications or herbal products affecting cervicitis (e.g., antibiotics, immunosuppressants, or vaginal medications within 2 wk before the study), and a history of Henna allergies.

### Intervention

All females in the intervention and placebo groups were treated with a 400 mg tablet of Cefixime, a 1 gr tablet of azithromycin daily, and metronidazole 500 mg twice daily for 5 days. In addition to oral medication, the intervention group received Henna oil vaginal suppository, and the placebo group received a placebo (suppository without henna oil) once per night for a week. Moreover, the same antibiotic regimen was prescribed for the partners of the participants.

### Randomization and blinding

The design of the current study was a triple-blind, controlled paralleled clinical trial. Participants were randomly allocated into one of the groups of Henna oil vaginal suppository (intervention group) or placebo vaginal suppository with a ratio of 1:1. The process of randomization was carried out using the random allocation software. The pharmacist determined the medication and the placebo with different codes of A and B. Researchers, statistical analysts, and participants were not aware of whether A or B was medication or placebo. At each visit, participants were randomly assigned to groups A or B. While they did not know in which group, intervention or placebo were placed. The suppository was handed over to women in containers that were opaque with a similar shape and seal, based on the codes defined in advance. The tools and materials required to produce the placebo were similar to the medication except that the Henna oil, as an active ingredient, was removed from the placebo suppository. No detectable difference existed between the medication and placebo. Researchers and participants stayed blinded to the group allocation until the end of the study and data analysis.

### Product preparation

#### Plant collection

Henna plants were gathered from a region near Sistan and Baluchestan called Dalagan where Henna is native to it and cultivated. It was identified and approved by a botanist at the Herbarium Center of the Yazd School of Pharmacy (voucher number: SSU0069). The leaves were then separated from the plant and pulverized by an electric mill (Assan Toos Shargh, Iran), and passed through mesh 40.

#### Ingredients

The following materials were used as ingredients of the suppository in medications and placebo: polyethylene glycol 400 (PEG 400) (Merck, Germany), PEG 4000 (Merck, Germany), tween 80 (Merck, Germany), sesame oil (Shirreza, Yazd).

#### Quality control of plant 

The assessment of total ash and acid-insoluble ash adhered to the guidelines outlined in the Iranian Pharmacopeia (13). Simultaneously, an antimicrobial evaluation was conducted in accordance with the specifications in the United States Pharmacopeia (USP) (14). Microbial surveillance for Henna and placebo suppositories occurred at the Microbiology Laboratory of the Medical School of Shahid Sadoughi University in Yazd. The microbiological scrutiny targeted specific microorganisms, including *Staphylococcus aureus*, *Pseudomonas aeruginosa*, *Escherichia coli*, *Salmonella* spp, *Candida albicans*, mold, and yeast. The standard operating procedure followed the criteria set in USP32 - NF27 USP and National Formulary.

#### Henna oil preparation

Henna oil was made based on the procedure presented in the book of *Qarabadin*, which mentions the pharmaceutical methods of PM. 50 gr of Henna powder was immersed in 300 ml of distilled water overnight. The next day, it was heated on the heater for one hr at 90 C. Then, it was filtered using the Buchner funnel vacuum. In the next step, the aqueous extract was blended with an equal amount of sesame oil, and it was boiled to concentrate and vaporize the whole of the water content as much as possible (for 3 hr) (9).

#### Henna oil vaginal suppository and placebo preparation 

To reach higher adherence and satisfaction in women, the Henna oil and placebo were formulated as a suppository. The Henna oil suppository was prepared in advance in accordance with previous work on the optimization and standardization of this formulation. Based on this article, suppositories for medication and placebo using PEG 400, PEG 4000, tween 80, and Henna oil (only in the intervention group) (9). The Pharmacy School of Shahid Sadoughi University of Medical Sciences prepared both Henna and placebo suppositories.

#### Total phenolic content

The Folin-Ciocalteu method was employed to gauge the total phenolic content of the Henna extract. Gallic acid (GA) served as the standard, and the total phenol was expressed in mg of GA equivalents per individual suppository. Solutions with concentrations of 10, 20, 40, 60, 80, 100, and 200 µg/ml of GA were prepared and combined with 0.5 ml of a 10-fold diluted Folin-Ciocalteu reagent, followed by the addition of 0.4 ml of 7.5% sodium carbonate after 3–8 min. After maintaining the tubes at laboratory temperature for 30 min, the absorbance was measured at 760 nm using spectrophotometry. This process was repeated 3 times for each determination. The total phenolic content of the Henna extract was determined using the equation derived from a standard GA calibration curve.

### Measurements and outcomes

Prior to the initiation of this trial and subsequent to the confirmation of cervicitis through colposcopy and biopsy, all participants completed a questionnaire to gather demographic and medical history information, including age, number of pregnancies, number of children, abortion history, type of delivery, contraception method, and smoking habits. Subjects' weight was assessed using a digital scale (SECA, Hamburg, Germany) with light attire and bare feet, recorded to the nearest 0.1 kg. Standing height was measured using a wall-mounted stadiometer, accurate to the nearest 0.5 cm. Body mass index (BMI) was calculated using the formula: BMI= weight /height ${\;}^{-2}$ (kg/m²).

**Figure 1:**
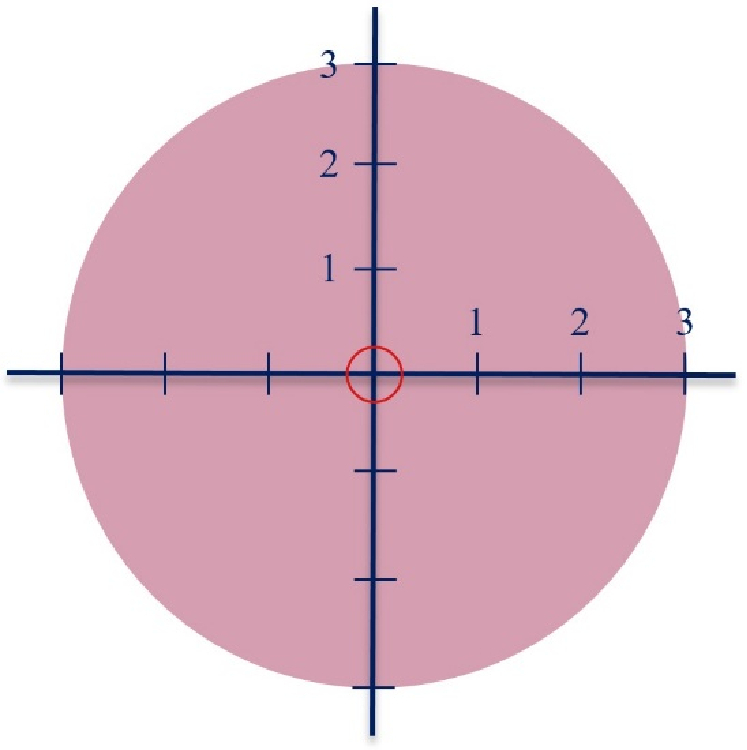
Range of cervical ulcer before and after the study.

The size and range of the ulcer were recorded by a regular gynecologist, visually, during a vaginal examination with a speculum before and 2 wk after the start of the study (Figure 1) (4). Moreover, fragility, which is one of the secondary outcomes, was also determined over these examinations using cotton swabs. It was identified by the presence or non-presence of bleeding upon palpating the cervix with a cotton swab.

Furthermore, additional outcomes were considered as secondary. The amount of discharge was estimated by patients' reports and clinical examination based on a visual analog scale ranging from 0–10. Vulvovaginal irritation was also measured according to a scale between 0 and 10 before and after intervention. Symptoms of dyspareunia and PCB were also identified as presence or absence that women reported as yes or no, respectively.

The appropriate time for taking the medication was at least 2 wk before the commencement of menstruation, and the participants were recommended to return for the next visit in the third week after the end of menstruation. In the case of menstruation, the appointment was put off until the closest time after it was ended.

Women were explained not to use any other medications for their illness during the treatment and in case of any problems, to contact the researcher. During the intervention, a questionnaire including potential side effects was completed by the women. To ensure the use of the drug and answer the possible questions of the attendant, a telephone call was made by the researcher in every 3 days. The contact number was also given to the women for any questions.

After 7 days, the individual was contacted, and symptoms were recorded. They were asked for a follow-up visit 7 days after the end of treatment. At the second visit, their symptoms, including discharge, irritation, dyspareunia, and PCB, were recorded. Participants were also examined by speculum; and the size of the cervical ulcer and fragility with the cotton swab were recorded. If a case did not improve after the intervention, she would receive the usual treatment prescribed by the gynecologist.

### Sample size

A prior similar study was applied to calculate the required sample size (15). Considering α = 0.05, power = 80%, d = 0.3, S^2^ = 0.215, and using the following formula, about 38 individuals were needed to be enrolled in each group. Finally, assuming a 20% loss, this overall figure was increased to 92 (n = 46/each group). 


$$N\geq \frac{{2(z_{1-\alpha /2}+z_{1-\beta })}^{2}s^{2}}{{\left(d\right)}^{2}}$$

### Ethical considerations

This research was confirmed by the Ethics Committee of Shahid Sadoughi University of Medical Sciences in Yazd, Iran (Code: IR.SSU.REC.1399.193). Furthermore, this intervention was registered in the Iranian Registry of Clinical Trials and was updated on April 10, 2024. The purpose of the project and its pros and cons were initially explained to the participants and written informed consent was taken from all participants before enrolment in the trial. Antibiotic therapy was administered to all females of both groups due to ethical issues (not deprived of standard treatment).

### Statistical analysis

The SPSS version 24.0 (SPSS Inc., Chicago, IL, USA) was applied to perform statistical analyses. The statistical significance was taken into account at the level below 5%. Continuous variables were presented as mean ± standard deviation (SD), while discrete variables were reported as frequency (percent). The Chi-square test was utilized to compare discrete variables, with Fisher's exact test employed when necessary. The normality of data distribution was evaluated using the Kolmogorov-Smirnov test. In intra-group comparison, the paired *t *test and Wilcoxon *t *test were applied for parametric and non-parametric variables, respectively. While, in inter-group analysis, independent sample *t* test was used for parametric variables and the Mann-Whitney U test for non-parametric variables.

## 3. Results

### Baseline characteristic

**Figure 2:**
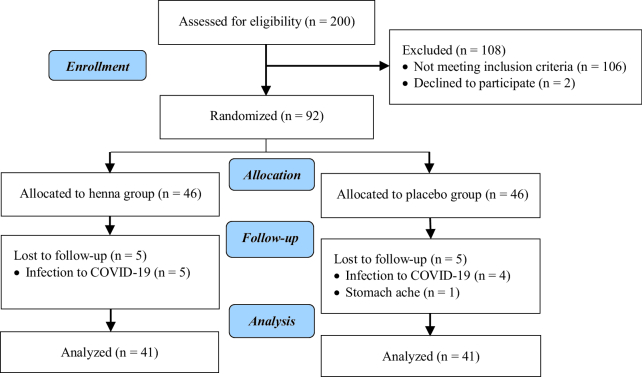
Flow diagram of the study.

Out of the 200 women referred for colposcopy and biopsy because of persistent vaginal discharge, PCB or menstruation issues, abnormal pap smear findings, or unusual cervix appearance during speculum examination, 106 participants were excluded based on positive human papillomavirus tests, presence of warts, dysplasia, or cancerous biopsy results. In addition, 2 cases refused to participate in the study. A total of 92 individuals whose biopsy results included cervicitis and had all inclusion and exclusion criteria, were enrolled in the study. During the study, 5 cases in the intervention group and 4 from the placebo group did not continue the intervention due to the coronavirus infection, and 1 case in the placebo group due to gastrointestinal complications of using antibiotics. Of those, 41 women in each group completed the study and were analyzed (Figure 2).

Demographic characteristics of the population have been shown in table I. No significant difference was observed at baseline features with respect to age, BMI, intercourse, pregnancy, abortion, number of children, type of delivery, past medical history of cervicitis, cervix freezing, smoking, method of contraception, and cause of referral. Therefore, these factors were comparable between groups before the commencement of the intervention.

### Quality control of herb

The total ash for Henna leaves was 10.891%, and acid insoluble ash was 2.941%. The total phenol content in the Henna extract was 171.561 μg/ml.

### Antimicrobial test

No clone was observed in any culture medium. The total amount of aerobic bacteria, molds, and fungi was zero, and the product was free of the 3 microorganisms *Pseudomonas aeruginosa*, *Staphylococcus aureus,* and *Candida albicans*.

### Efficacy evaluation

The results of quantitative variables of cervicitis symptoms between the Henna and the placebo group are shown in table II. At the baseline, no significant difference was observed between groups in terms of vaginal discharge (p = 0.96) and cervical ulcer size (p = 0.381), while vulvovaginal irritation was remarkable (0.042). At the end of the study, participants in the Henna group had a significantly lower score of vaginal discharge and cervical ulcer size (p < 0.001) in comparison with the placebo, and the difference between groups in the vulvovaginal irritation variable disappeared. In the within-group comparison, all variables changed significantly in both the intervention and placebo groups at the end of the study compared to the beginning (p < 0.001).

The results of the Chi-square test for qualitative variables of cervicitis symptoms did not demonstrate a significant difference in terms of dyspareunia (p = 0.658), PCB (p = 0.085), and friability (p = 0.635) before the intervention. At the end of the study, statistical significance was observed in variables of dyspareunia (p = 0.046) and PCB (p < 0.001), so no one in the intervention group had bleeding after intercourse. However, the difference in the friability variable between groups stayed insignificant (p > 0.999). In addition, when participants were compared to their baseline, all variables were improved in both groups, except for PCB which did not change significantly (Table III).

**Table 1:** Demographic characteristics of participants


**Variable**		**Henna oil suppository (n = 41)**	**Placebo (n = 41)**	**P-value**
**Age (yr)**				
	**21–30**	13 (31.7)	10 (24.4)	
	**31–40**	18 (43.9)	21 (51.2)	
	**41–50**	10 (24.4)	10 (24.4)	0.710*
**BMI (kg/m^2^)**				
	**< 18.5**	1 (2.4)	0 (0.0)		
	**18.5–25**	23 (56.1)	19 (46.3)	
	**25–30**	12 (29.3)	17 (41.5)	
	**30 <**	5 (12.2)	5 (12.2)	0.570**
**Intercourse (per week)**				
	**0**	8 (19.5)	8 (19.5)		
	**1–2**	26 (63.4)	23 (56.1)		
	**2 <**	7 (17.1)	10 (24.4)	0.860*
**Pregnancy**				
	**Zero**	3 (7.3)	3 (7.3)		
	**1 or 2**	23 (56.1)	20 (48.8)	
	**3 and more**	15 (36.6)	18 (43.9)	0.480**
**Abortion**				
	**Zero**	33 (80.5)	28 (68.3)		
	**1**	6 (14.6)	8 (19.5)	
	**2 and more**	2 (4.9)	5 (12.2)	0.370**
**Number of children**				
	**Zero**	4 (9.8)	4 (9.8)		
	**1**	9 (22.0)	3 (7.3)	
	**2**	16 (39.0)	23 (56.1)	
	**3 and more**	12 (29.2)	11 (26.8)	0.230**
**Type of delivery**				
	**No delivery**	4 (9.8)	4 (9.8)		
	**Cesarean delivery**	8 (19.4)	5 (12.2)		
	**Vaginal delivery**	25 (61.0)	27 (65.8)	
	**Both**	4 (9.8)	5 (12.2)	0.773**
**Past medical history of cervicitis**				
	**Yes**	40 (97.6)	38 (92.7)		
	**No**	1 (2.4)	3 (7.3)		0.610**
**Cervix freezing**					
	**Yes**	6 (14.6)	7 (17.1)	
	**No**	35 (85.4)	34 (82.9)	1.000*
**Smoking**				
	**Yes**	9 (21.9)	10 (24.4)	
	**No**	32 (78.1)	31 (75.6)	> 0.999*
**Method of contraception**				
	**Condoms**	8 (19.5)	7 (17.1)	
	**Oral contraceptive pill**	4 (9.8)	2 (4.9)	
	**Tubal ligation**	3 (7.3)	6 (14.6)	
	**IUD**	3 (7.3)	1 (2.4)	
	**No contraception**	23 (56.1)	25 (61.0)	0.590**
**Cause of refer**				
	**Discharge**	28 (68.3)	23 (56.1)		
	**Dyspareunia**	0 (0.0)	7 (17.1)		
	**Infertility**	2 (4.9)	0 (0.0)		
	**Periodic examination**	3 (7.2)	5 (12.1)	
	**Irritation and itching**	4 (9.8)	2 (4.9)	
	**AUB**	4 (9.8)	4 (9.8)	0.060**
Data presented as n (%). *Chi-Square test, **Fisher's exact test. BMI: Body mass index, IUD: Intrauterine device, AUB: Abnormal uterine bleeding				

**Table 2:** Intra and intergroup comparison of quantitative symptoms of cervicitis at the initial and final of the study


	**Before**			**After**			
**Variables**	**Henna**	**Placebo**	P-value*	**Henna**	**Placebo**	P-value*	P-value**
	**Vaginal discharge**	5.12 ± 2.18		5.14 ± 2.20	0.96			1.66 ± 1.24	3.75 ± 2.00	< 0.001	< 0.001
**Vulvovaginal irritation**	2.58 ± 2.71 (2.00, 5.00)	1.51 ± 2.25 (1.00, 1.50)	0.042	0.83 ± 1.26 (0.00, 2.00)	0.85 ± 1.37 (0.00, 1.00)	0.915	< 0.001
**Cervical ulcer size**	2.36 ± 1.26	2.17 ± 1.40	0.381	0.41 ± 0.64 (0.00, 1.00)	1.41 ± 1.33	< 0.001	< 0.001
Data presented as Mean ± SD. *Mann-Whitney test between groups, **Wilcoxon test before with after in each group. Data in parenthesis are median and interquartile range							

**Table 3:** Between comparison of qualitative symptoms of cervicitis at the initial and final of the study


**Variables**		**Henna**	**Placebo**	P-value*
**Dyspareunia**				
	**Before**	23 (56.1)	21 (51.2)	0.658
	**After**	4 (9.8)	11 (26.8)	0.046
	P-value**	< 0.001	0.002	
**Postcoital bleeding**				
	**Before**	8 (19.5)	15 (36.6)	0.085
	**After**	0 (0)	11 (26.8)	< 0.001
	P-value**	0.008	0.125	
**Friability**				
	**Before**	14 (34.1)	12 (29.3)	0.635
	**After**	5 (12.2)	5 (12.2)	> 0.999
	P-value**	0.004	0.016	
Data presented as number (percentage). *Chi-square test. **McNemar test				

## 4. Discussion

This study was a randomized clinical trial that assessed the effects of the Henna oil vaginal suppository without accompanying another herb on cervicitis. The results of this investigation indicated that the Henna oil vaginal suppository significantly decreased some symptoms of cervicitis, like vaginal discharge, cervical ulcer size, dyspareunia, and PCB, compared to the placebo.

Cervicitis, known as inflammation of the uterine cervix, is a disorder caused by infectious or non-infectious agents (6). As seen in this clinical trial, vaginal discharge, vulvovaginal irritation, and dyspareunia of cases in the Henna suppository group were remarkably reduced over the intervention. The efficacy of 2 vaginal creams containing Henna or Clotrimazole on women with vaginal candidiasis was also assessed, which the cream with Henna could be effective in improving some symptoms, including burning sensation, pruritus, discharge, and irritation of the vagina, and genital pain during intercourse. The study mentioned above also demonstrated that Henna vaginal cream lowers the candidiasis colonies and vaginal pH, while enhancing lactobacillus formation (16). Thus, it approves the anti-infectious activities of the Henna plant, which indicates this herb can be applied for infectious cervicitis. The antimicrobial effects of Henna can be attributed to the presence of naphthoquinone compounds like lawsone (17, 18), while it does not have the side effects of antibiotics. The *Lawsonia inermis* extract exhibited remarkable inhibitory properties against gram-positive and gram-negative bacteria such as *Escherichia coli*, *Salmonella typhi*, *Klebsiella* spp., *Shigella sonnei*, *Bacillus subtilis*, *Staphylococcus aureus*, *Staphylococcus epidermidis*, *Pseudomonas aeroginos*, *Bacillus cereus*, *Bacillus subtilis*, *Proteus vulgaris* (18). Additionally, Henna oil prepared based on the PM method had antimicrobial effects against *Gardnerella vaginalis*, *Neisseria gonorrhoeae*, and group B *Streptococcus *(9).

Moreover, the efficacy of oral *Sphaeranthus indicus* in combination with cream of *Lawsonia inermis* and *Plumbi oxidum* on symptoms of cervical erosion in women with cervicitis was investigated. The findings revealed that this polyherbal treatment is effective in the improvement of symptoms and healing of erosion. These results support the observations of the present trial, which indicated that the Henna oil reduced vaginal discharge, ulcer size of the cervix, dyspareunia, and PCB (19).

Painful intercourse, which is one of the symptoms of cervicitis, has negative effects on the quality of life mentally and physically (20). In agreement with our findings, the pain reliever effects of *Lawsonia inermis* were observed in people with chronic sciatica, where using a topical Henna formulation led to a significant decrease in pain severity and consequently promoted the quality of life (21). In addition, another trial demonstrated that a topical formulation of Henna resulted in a considerable improvement in contact dermatitis symptoms (22). These properties may be due to compounds derived from Henna, such as lawsone, isoplumbagin, and lawsaritol, which have anti-inflammatory and analgesic effects (23, 24).

Furthermore, the Henna suppository was significantly effective in the reduction of the size of cervical ulcers and PCB compared to the placebo group in the present research. Aligned with these results, a study found that Henna had protective effects in preventing and treating decubitus ulcers in critical care units (25). Another trial concluded that topical use of Henna is a practical treatment for epidermolysis bullosa by improving itching, burning, stringing, and cutaneous warmness sensation (26). It has been shown that Henna decreases epithelization time and accelerates wound contraction and healing by increasing collagen bands and fibroblasts and decreasing inflammatory cells (24, 27).

Some research has been conducted on the effect of traditional medicine formulations on females with cervicitis. In some of these formulations, Henna is one of their components (8, 9), but this study investigated the effects of Henna oil solely and as a vaginal suppository; so, this product could be prepared more easily and cost-effectively. For example, in a study that evaluated the effects of an oral drug along with a vaginal cream, where one of its ingredients was Henna, symptoms of cervicitis in the intervention group were completely improved while there were no signs of recovery in any of the cases in the placebo group (19).

In traditional medicine, including Persian and Chinese medicine, herbal compounds are sometimes used externally, including vaginal lavage, vaginal steaming, and intravaginal administration in the treatment of cervicitis and other infectious diseases of the female reproductive system (8, 28). It should be noted that topical use (local application) of the drug in these diseases is more effective than oral administration (29). In addition to the evidence that henna has anti-inflammatory properties, it also has other benefits such as availability, convenient use, inexpensiveness, improvement of symptoms and signs of cervicitis, non-invasiveness, and safety.

In the follow-up visit, the cases were only examined through observation, and due to the manipulation of the cervical tissue, it was not possible to perform a colposcopy again. For a better comparison of the antibiotic and the Henna suppository group, in the following works, only the Henna suppository can be prescribed. The polymerase chain reaction test was not used because of cost-effectiveness and expansiveness. In Iran, polymerase chain reaction is not routinely performed in all medical centers, and it is difficult for participants to have routine access to it. Moreover, cervicitis may be due to causes other than infectious agents.

## 5. Conclusion

The findings of the current trial indicated that using Henna oil vaginal suppositories, as a modality of complementary medicine, along with antibiotic treatments could improve vaginal discharge, cervical wound, dyspareunia, and bleeding after intercourse significantly in comparison with the placebo group. It is suggested that more studies be performed on the Henna suppository without antibiotic therapy and in a larger sample size.

##  Data availability

Data will be made available on request from the corresponding author.

##  Author contributions

N Nabimeybodi had full access to all of the data in the study and took responsibility for the integrity of the data and the accuracy of the data analysis. Concept and design: R Nabimeybodi, R Zareshahi, F Nokhostin, and M Kamalinejad. Acquisition, analysis, or interpretation of data: N Nabimeybodi, F Nokhostin, H Akhundimeybodi, N Seifi Mazraeno. Product preparation: N Nabimeybodi, R Zareshahi, and M Nabi Meybodi. Drafting of the manuscript: N Nabimeybodi, R Nabimeybodi. Critical revision of the manuscript for important intellectual content: All authors. Statistical analysis: F Madadizadeh. Supervision: R Nabimeybodi.

##  Conflict of Interest

The authors declare that there is no conflict of interest.
